# WELL-BEING IN NURSING HOME RESIDENTS THROUGH THE COVID-19 PANDEMIC

**DOI:** 10.1093/geroni/igad104.2022

**Published:** 2023-12-21

**Authors:** Emily Heilbrunn, Liza Behrens, Laura Felix, Erik Lehman, Nicole Osevala, Janice Whitaker, William Calo, Jennifer Kraschnewski

**Affiliations:** Penn State College of Medicine, Hershey, Pennsylvania, United States; Penn State Ross and Carol Nese College of Nursing, State College, Pennsylvania, United States; Penn State College of Medicine, Hershey, Pennsylvania, United States; Penn State College of Medicine, Hershey, Pennsylvania, United States; Penn State College of Medicine, Hershey, Pennsylvania, United States; Penn State Ross and Carol Nese College of Nursing, State College, Pennsylvania, United States; Penn State College of Medicine, Hershey, Pennsylvania, United States; Penn State College of Medicine, Hershey, Pennsylvania, United States

## Abstract

The SARS-CoV-2 (COVID-19) pandemic has disproportionately impacted residents in nursing homes. As a result of the vulnerable nature of congregate living, and to reduce resident morbidity and mortality, the Centers for Medicare and Medicaid Services (CMS) published Quality, Safety, and Oversight (QSO) guidance. Unfortunately, these restrictions may have had unintended negative consequences on the well-being of residents. Significant evidence illustrates that social isolation and loneliness have serious adverse health implications for older adults. We used GEE ordinal logistic regression modeling to examine the trends of depressive symptoms in nursing homes through the COVID-19 pandemic using the CMS Minimum Data Set (MDS). The PHQ-9 was leveraged to measure depressive symptoms, which was then transformed into a categorical variable (no depression = scores 0-4, mild depression = scores 5-9, moderate-severe depression = 10-27). We compared this with the 4-week incidence of COVID-19, transformed into a categorical variable (0 cases, 1-10 cases, 11-40 cases, >40 cases). The odds of depressive symptoms were elevated when comparing some cases to none (1-10 cases vs. 0 cases, OR 1.33 (1.31, 1.36), p-value < 0.001), 11-40 cases vs. 0 cases, OR 1.16 (1.14, 1.18), p-value < 0.001, >40 cases vs. 0 cases, OR 1.19 (1.18, 1.21), p-value < 0.001). While it is important to acknowledge that isolation-based practices are an effective means to reduce disease transmission, it is vital to recognize that they are in conflict with many quality-of-life goals. With this being said, nursing homes worked dutifully to protect residents’ health and well-being.

